# A Comparison of Intramuscular and Subcutaneous Administration of LigA Subunit Vaccine Adjuvanted with Neutral Liposomal Formulation Containing Monophosphoryl Lipid A and QS21

**DOI:** 10.3390/vaccines8030494

**Published:** 2020-09-01

**Authors:** Teerasit Techawiwattanaboon, Christophe Barnier-Quer, Tanapat Palaga, Alain Jacquet, Nicolas Collin, Noppadon Sangjun, Pat Komanee, Kanitha Patarakul

**Affiliations:** 1 Department of Microbiology, Faculty of Medicine, Chulalongkorn University, Pathumwan, Bangkok 10330, Thailand; teerasit.kku@gmail.com; 2 Chula Vaccine Research Center (Chula VRC), Center of Excellence in Vaccine Research and Development, Chulalongkorn University, Pathumwan, Bangkok 10330, Thailand; Alain.J@chula.ac.th; 3 Vaccine Formulation Laboratory (VFL), University of Lausanne, 1066 Epalinges, Switzerland; barnierquer@gmail.com (C.B.-Q.); nicolas.collin@unil.ch (N.C.); 4 Department of Microbiology, Faculty of Science, Chulalongkorn University, Bangkok 10330, Thailand; tanapat.palaga@gmail.com; 5 Armed Force Research Institute of Medical Sciences (AFRIMS), Ratchathewi, Bangkok 10400, Thailand; noppadon625@yahoo.com (N.S.); pat.afrims@gmail.com (P.K.)

**Keywords:** leptospirosis, *Leptospira*, LigA subunit vaccine, LMQ adjuvant, vaccination route, intramuscular, subcutaneous

## Abstract

Leptospirosis vaccines with higher potency and reduced adverse effects are needed for human use. The carboxyl terminal domain of leptospiral immunoglobulin like protein A (LigAc) is currently the most promising candidate antigen for leptospirosis subunit vaccine. However, LigAc-based vaccines were unable to confer sterilizing immunity against *Leptospira* infection in animal models. Several factors including antigen properties, adjuvant, delivery system, and administration route need optimization to maximize vaccine efficacy. Our previous report demonstrated protective effects of the recombinant LigAc (rLigAc) formulated with liposome-based adjuvant, called LMQ (neutral liposome combined with monophosphoryl lipid A and *Quillaja saponaria* fraction 21) in hamsters. This study aimed to evaluate the impact of two commonly used administration routes, intramuscular (IM) and subcutaneous (SC), on immunogenicity and protective efficacy of rLigAc-LMQ administrated three times at 2-week interval. Two IM vaccinations triggered significantly higher levels of total anti-rLigAc IgG than two SC injections. However, comparable IgG titers and IgG2/IgG1 ratio was observed for both routes after the third immunization. The route of vaccine administration did not influence the survival rate (60%) and renal colonization against lethal *Leptospira* challenge. Importantly, the kidneys of IM group showed no pathological lesions while the SC group showed mild damage. In conclusion, IM vaccination with rLigAc-LMQ not only elicited faster antibody production but also protected from kidney damage following leptospiral infection better than SC immunization. However, both tested routes did not influence protective efficacy in terms of survival rate and the level of renal colonization.

## 1. Introduction

Leptospirosis, caused by pathogenic *Leptospira* spp., is a neglected zoonosis affecting humans and animals mainly in poor sanitary and rural areas. The pathogenic bacteria may persist in asymptomatic carriers and cause chronic infection of the renal tubules in various wild and domestic animals. Humans may become ill after exposure to surroundings, especially water and soil, contaminated with urine from infected reservoirs [1,2]. Pathogenic leptospires enter across broken skin or mucus membrane and subsequently penetrate and disseminate hematogenously to target organs resulting in multiple organ dysfunction, such as tubulointerstitial nephritis, jaundice, liver failure, pulmonary hemorrhage, and myocarditis [1,3].

Although inactivated whole-cell vaccines for leptospirosis are commercially available, they have not been widely acceptable for human use because they confer short-term immunity, restrict cross-protection among pathogenic serovars, and induce several adverse effects such as local edema, pain, and fever [4]. Subunit vaccines have been developed to overcome the limitations of whole-cell vaccines. The variable carboxy-terminal domain 7–13 of leptospiral immunoglobulin-like protein A (LigAc) is currently the most promising antigen for leptospirosis vaccines [5,6,7]. Different types and formulations of LigAc-based vaccines, including DNA [8,9], recombinant single subunit [5,6,7], multisubunit [10,11], chimeric [12,13,14] vaccines, delivery systems [15], and adjuvants [16,17], have been tested for their efficacy in animal models. However, all these strategies were unable to completely prevent *Leptospira* renal colonization and pathological changes.

Intramuscular (IM) and subcutaneous (SC) administrations are common and convenient routes for mass vaccination in humans [18]. Previous studies showed that immunization routes had the effect on immunogenicity of vaccines. In humans, IM immunization of influenza vaccine yielded higher antibody titer than SC injection [19]. However, similar antibody titers were obtained between IM and SC routes in inactivated whole-cell leptospiral vaccine [20] and herpes zoster live-attenuated vaccine [21]. In mouse models, IM immunization with live-attenuated plague vaccine promoted faster antibody production and higher protection than SC injections [22]. Similar results were observed in hepatitis B surface antigen (HBsAg) adjuvanted with cationic-microsphere (MP) or alum [23]. IM vaccination with *M. tuberculosis* ESAT-6 and Ag85B proteins triggered high antibody titers, whereas the subunit vaccine remains poorly immunogenic once injected subcutaneously [24]. Taken together, the data indicated that administration route should be optimized to improve the vaccine efficacy.

Our previous study showed that recombinant LigAc (rLigAc) formulated with a liposome-based adjuvant called LMQ, composed of neutral liposome and two immunostimulants (MPL and QS21), conferred partial protection in hamsters [11] with overall 60% survival rate comparable with those obtained with rLigAc plus Freund’s adjuvant and alum [16,25]. To investigate the role of immunization route on the efficacy of rLigAc-LMQ, two common vaccination routes (IM and SC) were evaluated in the present study for the induction of rLigAc-specific antibody and the protective efficacy in hamsters, an animal model of acute leptospirosis.

## 2. Materials and Methods

### 2.1. Hamsters

Outbred golden Syrian hamsters were purchased from the North-East Animal Laboratory Center, Faculty of Medicine, Khon Kaen University, Thailand. All protocols involving manipulation of hamsters were approved by the Institutional Animal Care and Use Committee (IACUC)—the Armed Forces Research Institute of Medical Sciences (AFRIMS) Bangkok, Thailand (approval No. ARAC 1/60).

### 2.2. Leptospira Culture

The challenge experiments used low-passage *Leptospira interrogans* serovar Pomona [26]. Leptospires were cultured at 30 °C in Ellinghausen–McCullough–Johnson–Harris (EMJH) medium (Becton-Dickinson Difco™, MD, USA) supplemented with 10% bovine serum albumin (BSA) solution [27].

### 2.3. LMQ Preparation

A liposome (2.5 mg/mL cholesterol and 10 mg/mL 1, 2-dioleoyl-sn-glycero-3-phosphocholine) was produced by lipid film-rehydration and downsized by extrusion. The solutions of monophosphoryl lipid A (MPL) from *Salmonella enterica* serotype Minnesota (Sigma, MO, USA) and *Quillaja saponaria* fraction 21 (QS21) were mixed with the liposome suspension in a 1:3 *v*/*v* ratio. The final volume ratio of LMQ to immunogen was 6:4.

### 2.4. Recombinant LigAc Protein Production

The rLigAc was produced as described previously [11]. Briefly, inclusion bodies were isolated by centrifugation, washed with Tris buffer, pH 8.0 (50 mM Tris and 200 mM NaCl) containing 0.5% Triton X-100 and 1 M urea at 4 °C for 3 h, and solubilized in Tris buffer containing 6 M urea and 5 mM DTT overnight at 4 °C. The extracted proteins were purified by Ni^2+^ Chelating Sepharose column (GE Healthcare, Buckinghamshire, UK) under denaturing conditions. Purified rLigAc was refolded by dialysis with Tris buffer containing stepwise decreasing concentrations of urea (5 to 0 M). The secondary structure of purified rLigAc was evaluated by Jasco J-815 Circular Dichroism (CD) Spectropolarimeter (Jasco Incorporated, MD, USA) and analyzed with *CDPro* software. The factor H binding activity of the purified rLigAc was evaluated as described previously [11].

### 2.5. SDS-PAGE and Western Blotting

The purity of rLigAc was analyzed by 12% sodium dodecyl sulfate-polyacrylamide gel electrophoresis (SDS-PAGE) and stained with Coomassie brilliant blue R-250 (Bio-Rad, Germany). The Western blot detection of rLigAc was performed by mouse anti-6× His tag monoclonal primary antibody (1:5000, KPL, MD, USA) and goat antimouse alkaline phosphatase (AP)-conjugated secondary antibody (1:5000, KPL). The immunoreactivity was detected using BCIP/NBT Phosphatase Substrate Kit (KPL).

### 2.6. Immunization and Challenge

Groups of 5 female hamsters at 4–6 weeks of age were immunized with various vaccine formulations listed in Table 1. Hamsters were immunized three times at 2-week interval and blood samples were collected 1 week after the second and the third vaccination directly from the saphenous vein. Two weeks after the last immunization, hamsters were intraperitoneally challenged with 20× LD50 (200 cells) of virulent leptospires. The hamsters were weighted and monitored daily for moribund symptoms as previously described [5]. The hamsters that presented any of the endpoint criteria or survived up to 4 weeks postchallenge were euthanized.

### 2.7. Histopathology Determination

Tissue samples from kidney, lung, and liver were fixed in 10% formalin solution, embedded in paraffin, sectioned at 5 µm, and stained with hematoxylin and eosin. The histopathological examination was performed in blinded manner to reduce bias by a board-certified veterinary pathologist using a previously described grading system [16].

### 2.8. Detection of Viable Leptospires

Approximately, 100 µL of blood samples were inoculated into semisolid EMJH medium (0.2% agar). About half of each kidney sample was sliced into small pieces, pulverized by passing through 5 mL syringe, and inoculated into semisolid EMJH medium.

### 2.9. Quantitative Real-Time PCR (qPCR)

The genomic DNA from, approximately, 30 mg of kidney samples were extracted by TissueLyser LT (Qiagen) with QIAamp Fast DNA Tissue Kit (Qiagen) according to the manufacturer’s instruction. The qPCR was performed by QuantStudio 5 Real-Time PCR System (Applied Biosystem, CA, USA) with SsoAdvanced™ Universal SYBR Green Supermix (Bio-Rad) using specific primers for *lipL32* gene [28]. Leptospiral DNA standard curve was constructed from 10-fold serially diluted DNA of *Leptospira* equivalent to 2 × 10^9^ to 2 × 10^1^ cells/mL.

### 2.10. Enzyme-Linked Immunosorbent Assay (ELISA)

Each well of 96-well microtiter plates (Nunc, MA, USA) was coated with either rLigAc or recombinant LipL32 (rLipL32) protein (500 ng), leptospiral whole-cell lysates (1 × 10^6^ cells), or BSA (500 ng) overnight at 4 °C. The coated plates were blocked with blocking buffer (1% BSA in PBS plus 0.05% Tween 20, PBST) before addition of serially diluted hamster sera (1:100 to 1:312,500). The plates were incubated with 1:5000 horseradish peroxidase (HRP)-labeled goat antihamster IgG antibody (KPL). All incubation steps were performed at 37 °C for 1 h. After each incubation, the plates were washed five times with PBST. The reactivity of sera to the antigens was detected using TMB Substrate Set (BioLegend, CA, USA). The absorbance at 450 nm was measured by Varioskan Flash Spectral Scanning Multimode Reader (Thermo Scientific)

The same protocol was used to characterize levels of IgG subclasses with the exception that biotin-conjugated mouse antihamster IgG1 or IgG2 antibodies (1:5000; BD Pharmingen, NJ, USA) and streptavidin—HRP (1:5000; BD Pharmingen) were used as a secondary antibody.

### 2.11. Statistical Analysis

The antibody titers, histopathology scores, and bacterial burden were analyzed by Mann–Whitney U test. The survival curve was plotted using Kaplan–Meier method, and significant differences were determined by log-rank test.

## 3. Results

### 3.1. Preparation and Characterization of rLigAc

**Figure 1:**
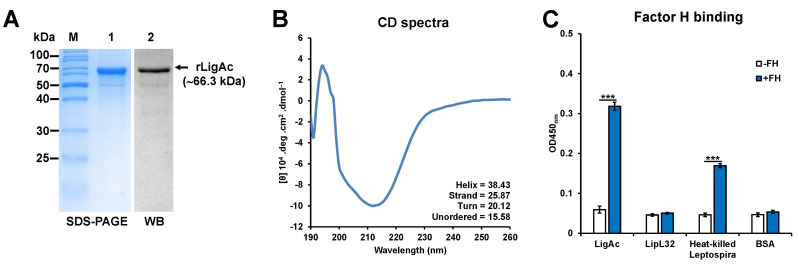
Analysis of the purified rLigAc by (**A**) Sodium dodecyl sulfate-polyacrylamide gel electrophoresis SDS-PAGE) and Western blotting (WB). Lane M = PageRuler™ Unstained Protein Ladder (Thermo Scientific); lane 1 = the rLigAc stained with Coomassie Brilliant Blue R-250; lane 2 = the rLigAc detected by WB with anti-6× His tag antibody. (**B**) Circular dichroism (CD) analysis. The CD spectra were measured by a JASCO J-815-150S spectropolarimeter and analyzed with *CDPro* software. The CD spectrum is represented as an average of more than five spectra from 190 to 260 nm. (**C**) Binding of rLigAc to purified human FH. The results are shown as mean ± SD absorbance at 450 nm. Student *t*-test was used to compare the absorbance between coated proteins; *** represents *p* < 0.001. Heat-killed *Leptospira* was used as a positive control, and rLipL32 and bovine serum albumin (BSA) were used as negative control.

His-tagged rLigAc was produced in *E. coli* as inclusion bodies. The protein was purified under denaturing conditions and then refolded by stepwise dialysis. The purified protein was used at a concentration of 1 mg/mL because precipitation occurred at higher concentration. The purity and secondary structure content of rLigAc were analyzed before vaccination. SDS-PAGE and immunoblotting with anti-6× His tag antibody confirmed the integrity of rLigAc, which can be detected as a band of 66.3 kDa corresponding to the predicted MW (Figure 1A). The CD spectra indicated that the purified rLigAc was refolded to its secondary structure (Figure 1B). Moreover, the ability to bind to human FH (Figure 1C) suggested that the purified rLigAc retained its FH binding activity.

### 3.2. Immunogenicity of rLigAc-LMQ in Hamsters

**Figure 2:**
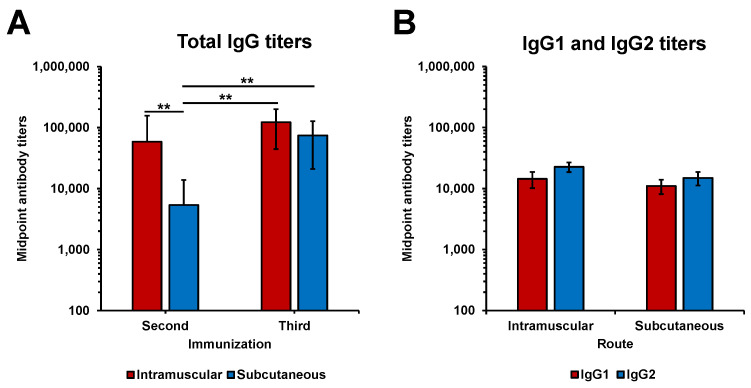
Antibody levels in the vaccinated hamsters. (**A**) Total LigAc-specific IgG titers measured 1 week after the second and the third immunizations. (**B**) Specific IgG1 and IgG2 titers measured at 1 week after the third immunization. The results are shown as mean ± SD. Mann–Whitney U test was used to compare antibody titers or IgG subclasses between groups; ** represents *p* < 0.01.

LigAc-specific antibody levels after the second and the third immunizations in hamsters were measured by ELISA. Total specific IgG titers following immunizations with rLigAc-LMQ via IM and SC routes were significantly higher than the undetectable antibody level in the control group after the second immunization. The third SC immunization induced higher antibody titers than the second one, while two IM immunizations were enough to develop the highest antibody level (Figure 2A). More importantly, two IM vaccinations triggered significantly higher antibody titers than two SC administrations (*p* < 0.01). However, the antibody titers were comparable in IM and SC groups after the third immunization. Anti-LigAc IgG titers in the HK group was significantly lower than those in rLigAc-LMQ groups immunized via IM and SC routes (Supplementary Figure S1). This result indicated that LigAc was unlikely responsible for protection in the HK vaccine. In addition, the anti-LigAc and anti-*Leptospira* IgG titers in hamsters immunized with either HK or rLigAc-LMQ vaccines were detected as shown in Supplementary Figure S2. Anti-LigAc IgG titers in the HK group was significantly lower than those in the IM and SC rLigAc groups. In contrast, anti-*Leptospira* titers in the HK group was significantly higher than those in the IM and SC rLigAc groups. These results indicated that complete protection conferred by the HK vaccine was not due to higher titers of IgG. As expected, the reactivity of tested sera to LipL32, a recombinant 6× His tag unrelated protein was not detected (Supplementary Figures S3 and S4). The IgG responses conferred by either route were both IgG1 and IgG2, which were not significantly different (Figure 2B). The specific IgG2/IgG1 ratio was 1.58 and 1.36 in the SC and IM groups, respectively.

### 3.3. Protective Efficacy of rLigAc-LMQ in Hamsters

**Figure 3:**
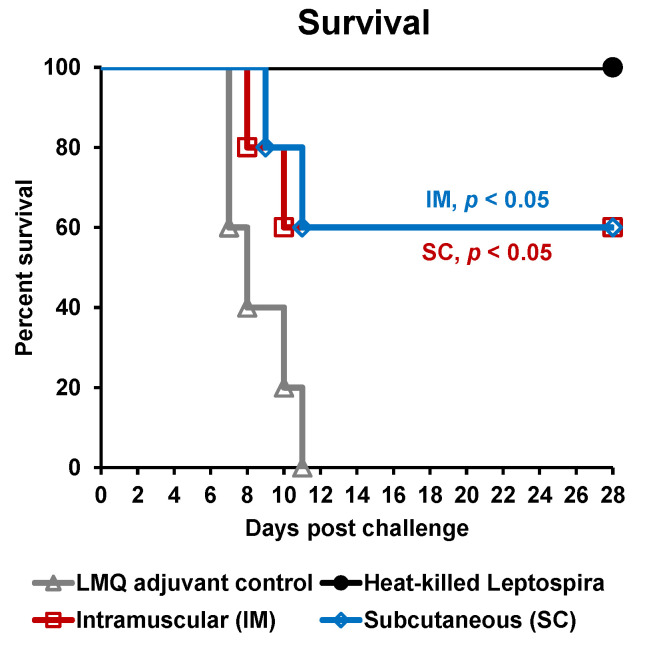
Kaplan–Meier plot of survival rates in vaccinated hamsters (n = 5 per group) following lethal challenge by virulent *Leptospira*. The hamsters were immunized with various vaccine formulations shown in Table 1. Each vaccinated hamster was challenged by 20× LD50 of low passage leptospires. The percent survival was calculated as the number of survivors/total challenged hamsters ×100. Statistical analysis of survival rates between control group and other vaccinated groups was performed by log-rank test.

The effect of vaccination with rLigAc-LMQ on survival, histopathological changes, and leptospiral burden in kidneys were evaluated in a hamster model of lethal leptospirosis. The rLigAc-vaccinated hamsters, whatever the route used, presented the same 60% survival rate compared to 0% survival in the control group (*p* < 0.05) following the challenge (Figure 3 and Table 2). All nonsurviving hamsters presented at least one of the endpoint criteria. Two out of five hamsters from each control and SC group lost their body weight over 10% on day 11 after challenge and were euthanized. As expected, the hamsters vaccinated with the killed whole cell vaccine showed 100% survival after challenge [11].

The necropsy of all surviving hamsters demonstrated various degrees of organ lesions (Table 2 and Supplementary Figure S5). Few foci of liver inflammation were found in all survivors except for three out of five hamsters vaccinated with the killed vaccine which showed no lesions. Mild to moderate lesions with small foci of lung hemorrhage were detected in all survivors. The mean pathology score of lungs in the hamsters received rLigAc by IM and SC routes was significantly higher than those received the killed vaccine (*p* < 0.05 and *p* < 0.01, respectively). However, liver and lung pathologies were not significantly different between IM and SC groups. Interestingly, tubulointerstitial nephritis indicating renal injury was found only in surviving hamsters from the SC group but not the IM group (*p* < 0.05). Like the IM group, no pathological change was found in the kidneys of surviving hamsters from the HK group.

**Figure 4:**
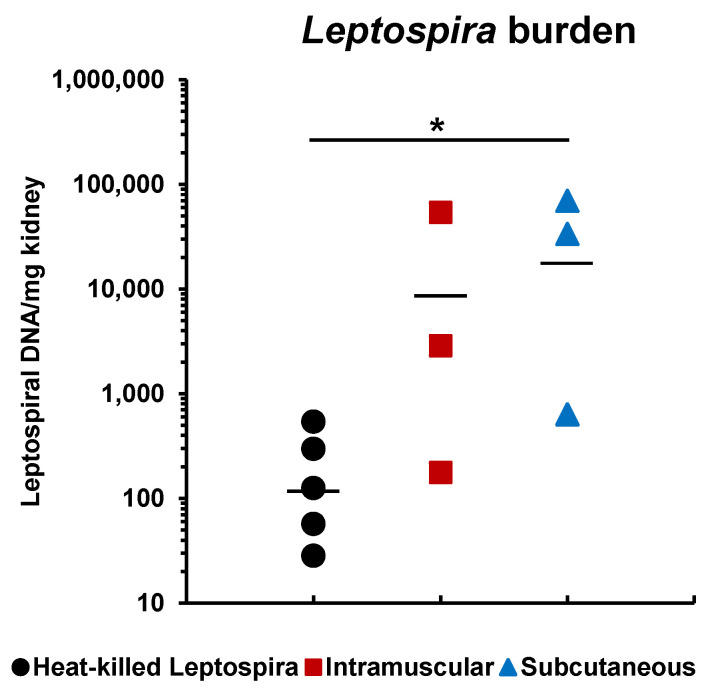
Leptospiral burden in the kidneys of surviving hamsters after challenge. The leptospiral genome was detected by qPCR. The cycle threshold of each sample was compared with leptospiral DNA standard curve to calculate bacterial load, which is expressed as bacterial DNA per milligram of tissue. Mann–Whitney U test was used to compare bacterial number among vaccination groups; * represents *p* < 0.05.

Blood cultures for leptospires of all surviving hamsters were negative (Table 2). Viable *Leptospira* were detected in the kidneys of two surviving hamsters immunized with the rLigAc via both routes and one hamster immunized with the killed vaccine. Interestingly, the leptospiral burden quantified by qPCR in the kidneys of surviving hamsters immunized with the rLigAc vaccine via SC injection were significantly higher than those received the killed vaccine (*p* < 0.05) (Figure 4). Renal colonization in hamsters vaccinated subcutaneously seems to be higher than that in the IM group, but the difference was not statistically significant.

## 4. Discussion

In the last decade, development of leptospirosis vaccines has been focused on subunit vaccines to overcome the drawbacks of commercially available killed whole-cell vaccines [4,29]. LigAc is currently considered as the most promising antigen for leptospirosis subunit vaccines as it conferred high level of protection (60–100% survival) in animal models [5,6,7]. Several LigAc-based vaccines using multiple platforms, such as DNA, protein, chimeric vaccines, were employed [4,29]. LigAc subunit vaccines have been formulated with various adjuvants including Freund’s, alhydrogel, liposome, xanthan gum, PLGA, and *Salmonella* flagellin (FliC) [5,6,7,10,16,17,30]. However, none of LigAc-based vaccine formulations induced sterilizing immunity and completely prevented renal colonization and kidney damage in hamster models. In our previous study, rLigAc (20 µg) adjuvanted with LMQ also induced partial protection in hamsters [11] at a similar level in comparison to rLigAc formulated with Freund’s adjuvant and alum [7,16].

The administration route of vaccination can influence the vaccine immunogenicity and efficacy because the vaccine localization is determinant for efficient priming of immune cells and subsequently optimal local and systemic immune responses [31]. For example, IM immunizations of mice with live-attenuated plaque or tuberculosis subunit vaccine induced faster antibody production than the SC route [22,32]. In this study, we aimed to compare immunogenicity and protective efficacy of rLigAc-LMQ vaccine via IM and SC administrations. We selected these two parenteral routes as they were compatible with our vaccine formulation and systemic immune response was required for protective immunity against intraperitoneal *Leptospira* challenge.

Our present study showed that IM administration of rLigAc-LMQ vaccine induced faster antibody production than SC route because the highest antibody titers were already reached in the IM group after the second injection, but three SC immunizations were needed to obtain the same antibody level (Figure 2A). Different kinetics of antibody responses observed with IM and SC routes are likely associated with a variation in anatomical and physiological conditions and access to cellular innate immune cells at the vaccination sites. Vaccine antigen is first recognized by local and infiltrating innate immune cells at the injection site before the antigen presentation to stimulate adaptive immunity [33]. Therefore, the unique repertoire of innate immune cells at the injection site may greatly influence the adaptive immune responses. Subcutaneous tissue is poor in antigen-presenting cells (APCs) [34], so the SC administration may not be optimal for efficient antigen presentation. In contrast, abundant blood and lymphatic capillaries in muscle tissue may facilitate immune cell trafficking between sites of vaccination and draining lymph nodes [35]. Thus, IM administration can possibly deliver vaccine antigens and stimulate immune responses more rapidly than SC route [36] as shown in our results.

Previous reports in mouse models demonstrated that immunoglobulin class-switching is dependent not only on vaccine formulation but also on immunization route [23,37]. In our study, the IgG subclass profiles triggered by both parenteral immunizations were not different, indicating that route of vaccination did not affect the IgG isotype switching of hamsters after immunizations with rLigAc-LMQ (Figure 2B). The pattern of IgG isotype was consistent with our previous findings showing that the rLigAc and LMQ formulation stimulated balanced Th1/Th2 immunity in hamsters [11]. This result was expected as MPL and QS21 could strongly induce production of both IgG1 and IgG2 (Th2 and Th1-biased antibodies, respectively) [38,39].

Leptospirosis vaccines with high protective efficacy and lower side effects are required for human use. In healthy volunteers, IM administration of inactivated vaccines of *L. interrogans* caused less frequent local reactions than when injected subcutaneously. However, the antibody responses were shown to be similar, whatever the route of injection [20]. In different preclinical studies, rLigAc subunit vaccines were mostly delivered to animal models via SC route [5,6,7,10,11,16,17,30], while only one experiment evaluated IM immunizations [40]. It is difficult to compare the results produced by these studies because of discrepancies in vaccine formulations, administration routes, and *Leptospira* strain and challenge dose. In this study, IM and SC routes for the rLigAc-LMQ vaccine demonstrated equivalent protective efficacy in terms of survival (Figure 3 and Table 2). It is not surprising because before challenge, the rLigAc-specific antibody levels in both IM and SC groups were comparable after three doses of vaccination. So far, the antibody level and immune response against rLigAc correlated to protection is not known, thus we cannot predict the protective efficacy after two doses of immunization via SC route, which induced lower antibody level.

Interestingly, no kidney pathological changes were observed in the surviving hamsters vaccinated through IM route (Table 2 and Supplementary Figure S5). The statistical analysis demonstrated the kidney pathology score was correlated with early LigAc-specific antibody titers after the second immunization (*R*^2^ = 0.878) better than those after the third immunization (R^2^ = 0.488) (Supplementary Figure S4). The surviving hamsters immunized with rLigAc-LMQ via IM route showed lower histopathological score than the SC group might be associated with higher LigAc-specific IgG titers after the second immunization (Figure 2A). Our results agree with the previous report by Lourdault and colleagues showing the correlation (R^2^ = 0.79, Pearson) between protective efficacy of *E. coli* expressing LigAc oral vaccine and LigAc-specific antibody levels at the early stage in the immunization process [15]. In our study, LigAc-specific antibody in the rLigAc-LMQ via IM group reached peak levels (after the second immunization) earlier than the SC group (after the third immunization). It is possible that B cells in the IM group undergone affinity maturation earlier resulting in LigAc-specific IgG with better affinity than that in the SC group [41]. However, the role of IgG isotypes and T cells in prevention or recovery of pathological changes cannot be excluded. However, renal colonization by *Leptospira* was still detected in the IM and SC groups (Figure 2B), indicating that sterilizing immunity and complete protection against *Leptospira* infection could not be achieved via either route. Therefore, under our experimental settings, the administration route had no obvious impact on efficacy and prevention of renal colonization of the rLigAc-LMQ vaccine formulation.

Appropriate vaccination schedule together with minimal number of doses to achieve high immune response and maximal protection are desirable to promote animal welfare in preclinical studies, reduce cost and side effects, and improved compliance for human use. In our study, two IM injections with rLigAc-LMQ (20 µg of rLigAc) at 2-week intervals should be sufficient to achieve adequate antibody level and protection for further studies in a hamster model. However, a total volume of each vaccine dose is another factor to select appropriate administration route because SC injection site can generally be delivered 5–10 times greater volume than IM site [42]. Knowledge obtained from this study will allow for the development of optimal vaccination strategies to maximize immune responses and protective efficacy of vaccines in preclinical and clinical studies in the future.

## 5. Conclusions

Optimal route of administration was evaluated as one of vaccine strategies for the rLigAc-LMQ vaccine formulation to obtain acceptable vaccine immunogenicity and protective efficacy. Our data demonstrated that IM administration induced antibody production faster than SC route as shown by significantly higher antibody titers in the IM group after the second immunization. After three doses of immunizations, antibody levels were comparable with a similar pattern of balanced IgG1 and IgG2 in both routes. Although no pathological changes in the kidneys were observed in the surviving hamsters vaccinated via IM route, both IM and SC groups showed no difference in terms of protective efficacy and renal colonization against lethal challenge in hamsters.

## Figures and Tables

**Table 1:** Experimental design and vaccine formulations.

Group	Antigen	Dose	Adjuvant	Volume	Route
Control	Tris buffer	–	LMQ	250 µL	Subcutaneous
HK	Heat-killed *Leptospira*	10^8^ cells	Freund’s	250 µL	Subcutaneous
IM	rLigAc	20 µg	LMQ	75 µL in each hind leg	Intramuscular
SC	rLigAc	20 µg	LMQ	250 µL	Subcutaneous

**Table 2:** Protective efficacy conferred by different vaccine formulations.

Group ^a^	Protection ^b^	Endpoint Days	Positive Culture ^c^		Pathology Score ^d^		
			Blood	Kidney	Lung	Liver	Kidney
Control	0%	7, 7, 8, 10, 11	ND	ND	ND	ND	ND
HK	100% **	28, 28, 28, 28, 28	0/5	1/5	1, 1, 1, 1, 1	0, 0, 0, 1, 2	0, 0, 0, 0, 0
IM	60% *	8, 8, 28, 28, 28	0/3	2/3	1, 2, 2 *	0, 1, 2	0, 0, 0
SC	60% *	9, 11, 28, 28, 28	0/3	2/3	2, 2, 2 **	1, 1, 1	1, 1, 1 **^,#^

^a^ HK = heat-killed *Leptospira*; IM = intramuscular; SC = subcutaneous. ^b^ The % protection was calculated as the number of survivors/total challenged hamsters × 100. Statistical analysis of survival rate between the control group and other vaccinated groups was analyzed by log-rank test; * represents *p* < 0.05 and ** represents *p* < 0.01. ^c^ Leptospiral culture was performed only in the surviving hamsters. The results show the number of positive culture/total surviving hamsters. ^d^ The pathological scores were determined only in the surviving hamsters. Pulmonary hemorrhage and tubulointerstitial nephritis were graded as 0–3 (none–severe). Liver pathology was graded based on the average number of inflammatory foci in 10 fields at 10× magnification as 0 (none), 1 (1–3), 2 (4–7), or 3 (>7). Mann–Whitney U test was used to compare statistical values of pathological score between HK group and other vaccination groups; * represents *p* < 0.05; ** represents *p* < 0.01; and between IM and SC rLigAc vaccination groups, # represents *p* < 0.05.

## Supplementary Materials

The following are available online at https://www.mdpi.com/2076-393X/8/3/494/s1, Figure S1: Total anti-LigAc IgG antibody titers in the vaccinated hamsters; Figure S2: Antibody levels in the vaccinated hamsters; Figure S3: The reactivity of hamster sera to rLigAc, rLipL32, and BSA; Figure S4: The reactivity of hamster sera against rLigAc and rLipL32; Figure S5: Histopathology of lung, liver, and kidney in the surviving hamsters vaccinated with various vaccine formulations (Table 1); and Figure S6: Statistical analysis of correlation between total LigAc-specific IgG titers, pathology score, and leptospiral burden of surviving hamsters in IM and SC LigAc groups.
