# Supplementary material for: A Comparison of Intramuscular and Subcutaneous Administration of LigA Subunit Vaccine Adjuvanted with Neutral Liposomal Formulation Containing Monophosphoryl Lipid A and QS21
Source: Vaccines (Basel). 2020 Sep 1;8(3):494. doi: 10.3390/vaccines8030494 (PMC7565420; doi:10.3390/vaccines8030494)
Supplement: Supplementary file 1 [file vaccines-08-00494-s001.pdf]

Figure S1. Total anti-LigAc IgG antibody titers in the vaccinated hamsters. Total anti-LigAc IgG titers in hamsters immunized with HK vaccine or rLigAc vaccine immunized via intramuscular and subcutaneous route were measured one week after the second and the third immunizations. The results are shown as mean ± SD. Mann–Whitney *U* test was used to compare antibody titers between groups at each time point; ** represents p < 0.01.

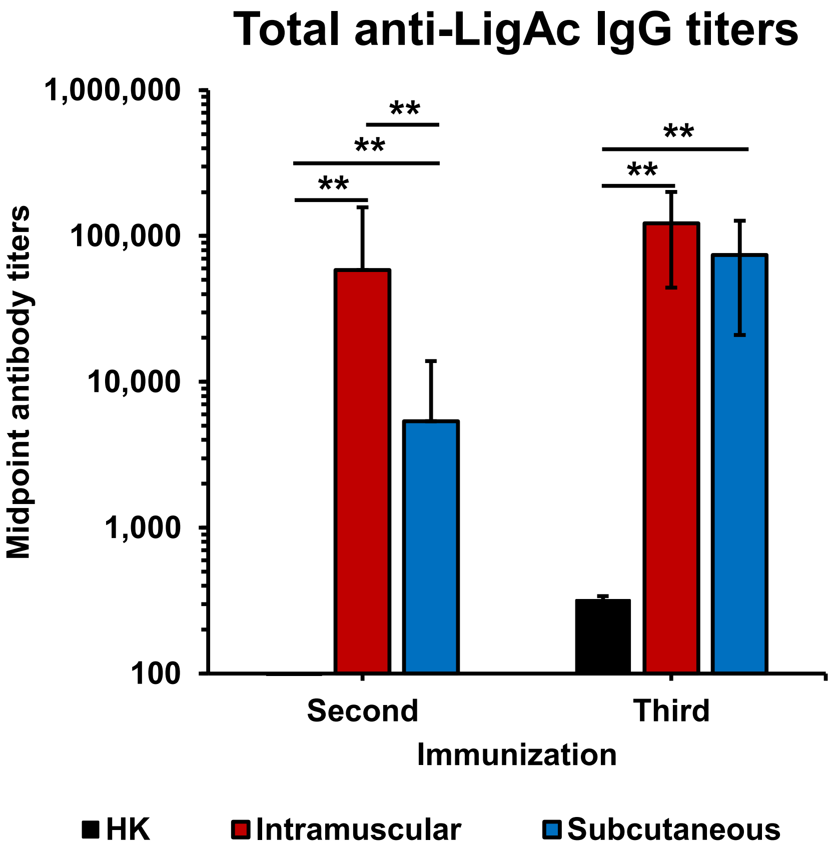

Figure S2. Antibody levels in the vaccinated hamsters. Total anti-Leptospira and anti-LigAc IgG titers in hamsters immunized with HK vaccine or rLigAc vaccine were measured one week after the third immunizations. The results are shown as mean ± SD. Mann–Whitney *U* test was used to compare antibody titers between groups; * represents $p < 0.05$ and ** represents $p < 0.01$.

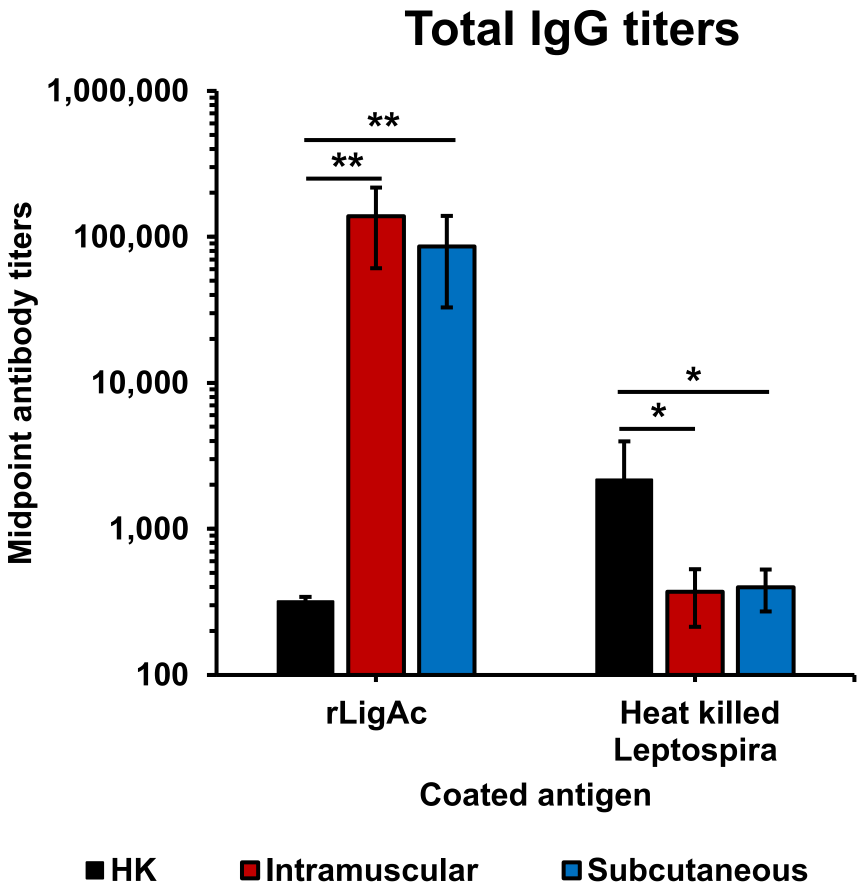

Figure S3. The reactivity of hamster sera to rLigAc, rLipL32, and BSA. The reactivity was detected by ELISA with TMB Substrate Set. The absorbance was measured by Varioskan Flash Spectral Scanning Multimode Reader. The result showed mean ± SD absorbance at 450nm.

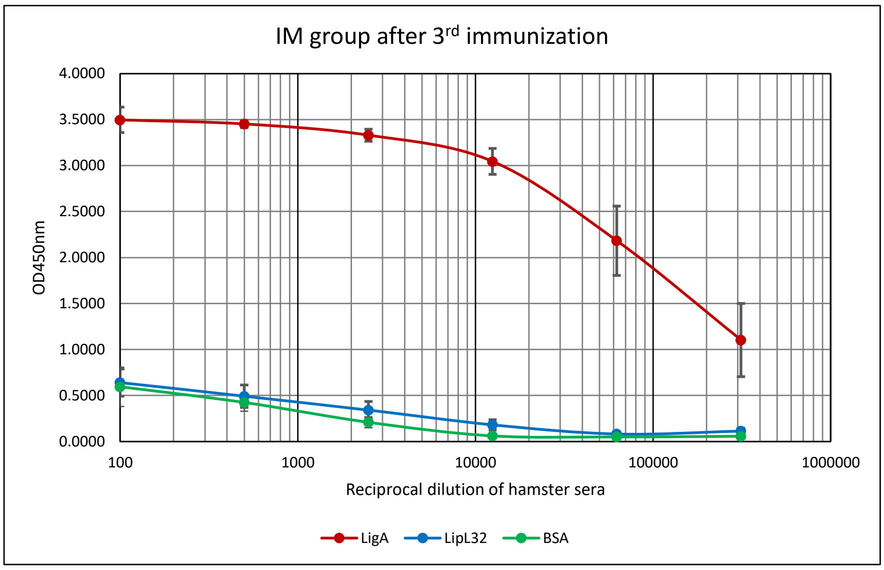

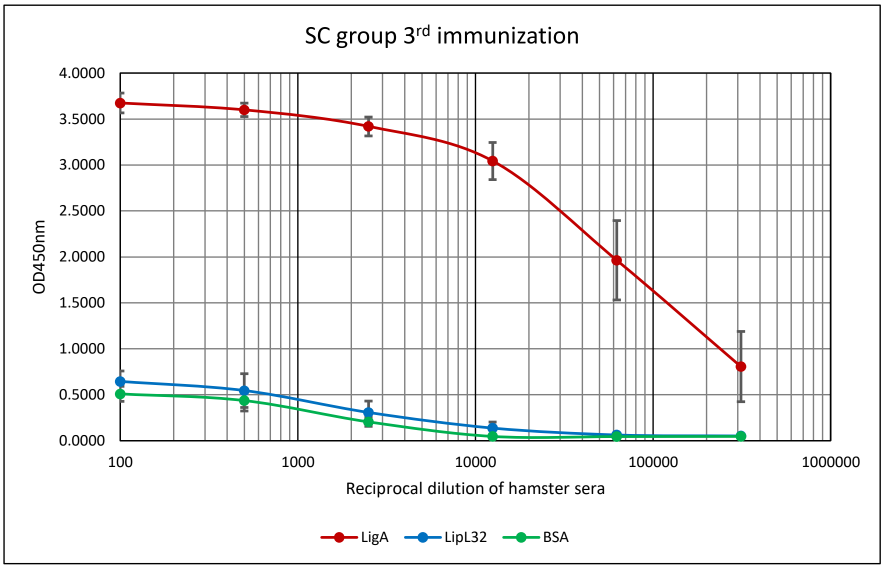

Figure S4. The reactivity of hamster sera against rLigAc and rLipL32. The rLigAc (1 μg to 0.1 ng) and rLipL32 (3 μg) were subjected to 12% SDS-PAGE and transferred to nitrocellulose membrane. The membrane was incubated with 1: 100 hamster serum after the third immunization (pooled sera of all hamsters in the IM and SC groups). The Western blot detection was performed with 1: 5,000 horseradish peroxidase (HRP)-labeled goat anti-hamster IgG antibody (KPL). The immunoreactivity was detected using Clarity and Clarity Max ECL Western Blotting Substrates (GE Healthcare). The images were captured by ChemiDoc XRS+ System (Bio-Rad).

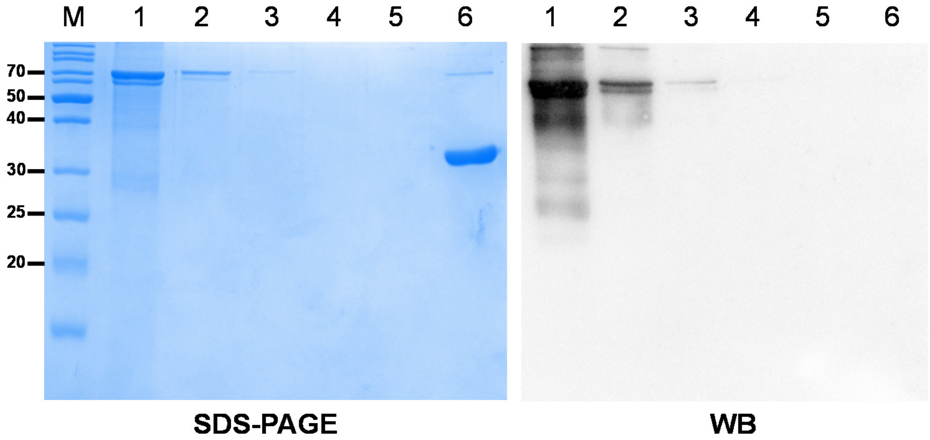

Lane	Protein
M	PageRuler™ Unstained Protein Ladder
1	rLigAc (1 μg)
2	rLigAc (100 ng)
3	rLigAc (10 ng)
4	rLigAc (1 ng)
5	rLigAc (0.1 ng)
6	rLipL32 (3 μg)

Figure S5. Histopathology of lung, liver and kidney in the surviving hamsters vaccinated with various vaccine formulations (Table 1).

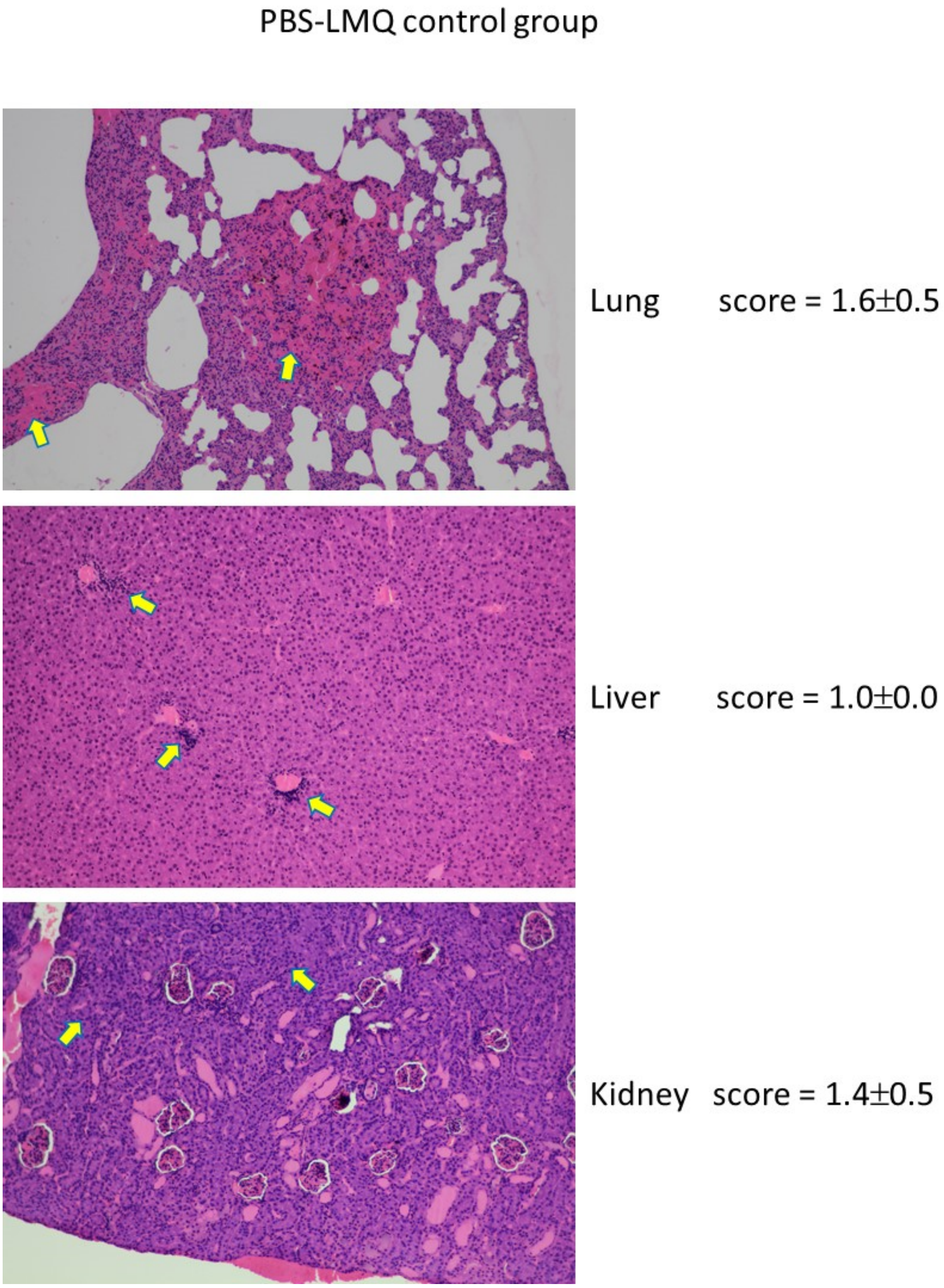

Heat-killed whole cell group

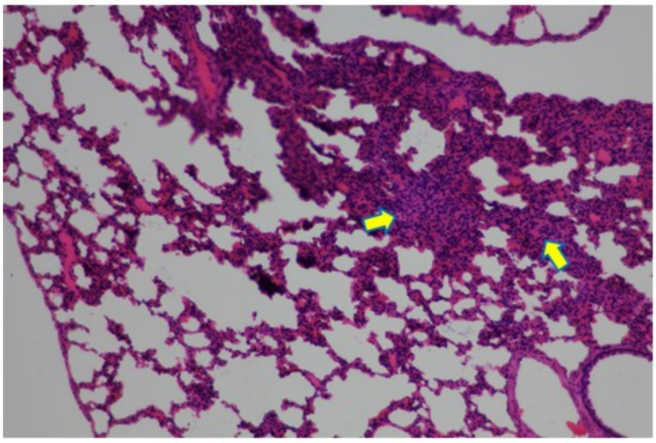

Lung score = 1.0±0.0

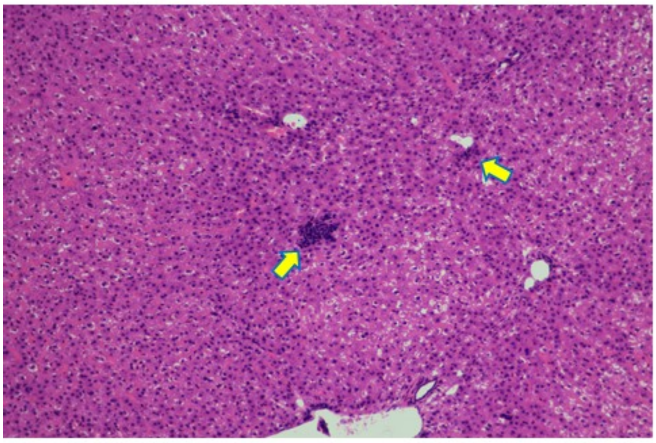

Liver score = 0.6±0.9

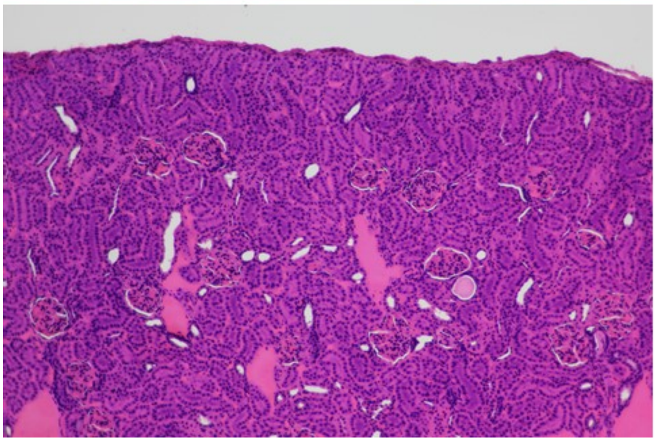

Kidney score = 0.0±0.0

Intramuscular group

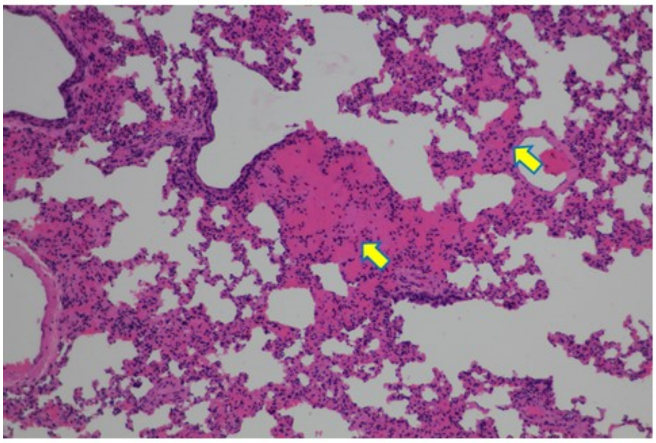

Lung score = 1.4±0.5

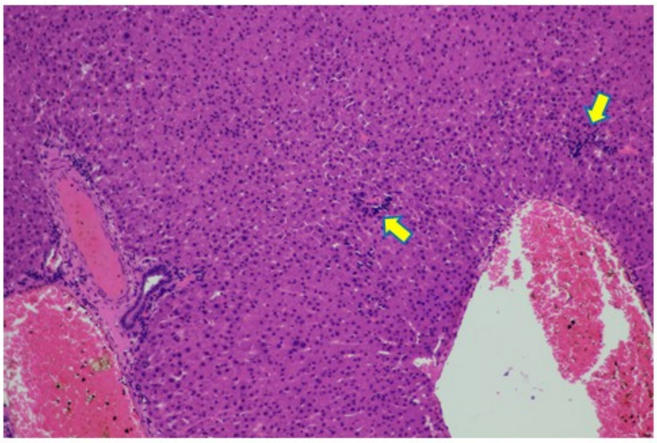

Liver score = 0.8±0.8

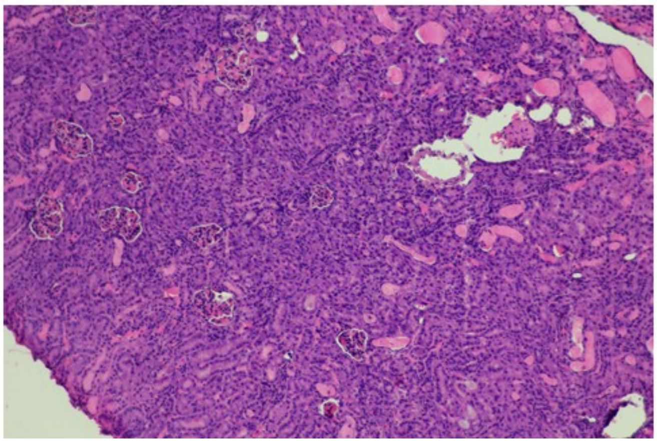

Kidney score = 0.0±0.0

Subcutaneous group

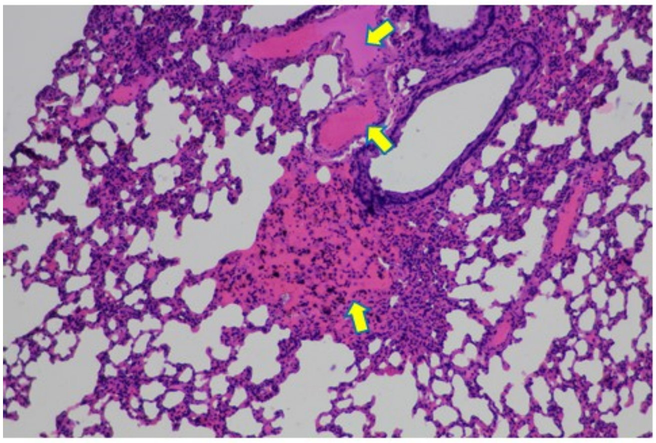

Lung score = 1.6±0.5

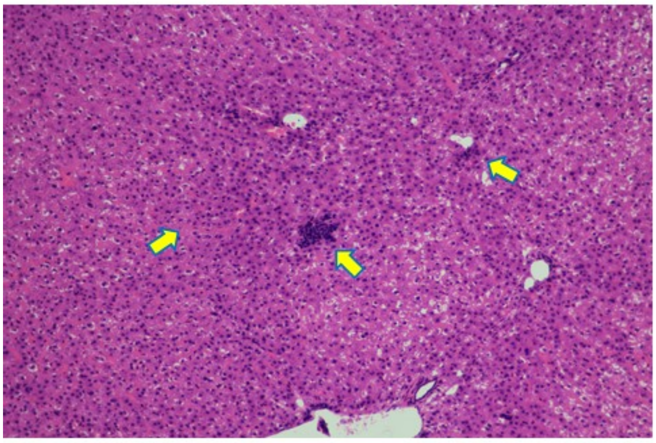

Liver score = 1.0±0.7

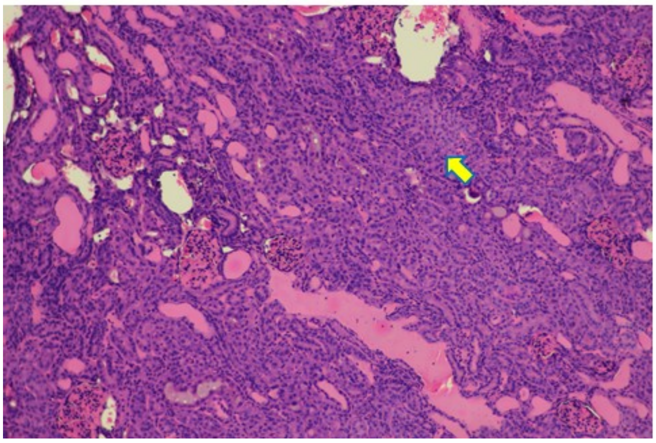

Kidney score = 0.8±0.8

Figure S6. Statistical analysis of correlation between total LigAc-specific IgG titers, pathology score, and leptospiral burden of surviving hamsters in IM and SC LigAc groups. The statistical analyses were performed using IBM SPSS Statistics for Windows (version 22, Chulalongkorn University license). The bivariate correlation with Spearman's rank correlation coefficient was used.

Correlations

			Abtiter2nd	Abtiter3rd	Pathology	Leptospiralload
Spearman's rho	Abtiter2nd	Correlation Coefficient	1.000	.829*	.878*	-.029
		Sig. (2-tailed)	.	.042	.021	.957
		N	6	6	6	6
	Abtiter3rd	Correlation Coefficient	.829*	1.000	.488	.086
		Sig. (2-tailed)	.042	.	.326	.872
		N	6	6	6	6
	Pathology	Correlation Coefficient	.878*	.488	1.000	-.293
		Sig. (2-tailed)	.021	.326	.	.573
		N	6	6	6	6
	Leptospiralload	Correlation Coefficient	-.029	.086	-.293	1.000
		Sig. (2-tailed)	.957	.872	.573	.
		N	6	6	6	6

*. Correlation is significant at the 0.05 level (2-tailed).
